# Martin Craig Downer (1931-2017): Chief Dental Officer, dental public health professor, editor, musician and novelist

**DOI:** 10.1038/s41415-024-8228-4

**Published:** 2025-04-25

**Authors:** Stanley Gelbier

**Affiliations:** Honorary Professor and Head of the Unit for the History of Dentistry, Faculty of Dentistry, Oral and Craniofacial Sciences, King´s College London, Guy´s Hospital, Tooley Street, London, SE1 1UL, United Kingdom

## Abstract

Professor Martin Downer was a leading public health dentist. He was an administrator, Chief Dental Officer of Kensington and Chelsea, and later to the Departments of Health in Scotland and England. Finally, he was Professor of Oral Health Policy at the Eastman Dental Institute. His research was wide-ranging, but he made a notable international mark with his work investigating oral cancer screening. Like many great men, there was another side to him; he was a musician and a saxophonist from his student days, which led to *Top of the Pops* and a recording studio. To top it all, he wrote novels.

## Key points

- It's a truism that public health dentistry in the United Kingdom grew from the school dental services, which provided experience in simple surveys, dental health education and so on.
- A basis of practice in the school and community services give administrative experience which is invaluable on the way to the top dental posts in the Scottish and English Departments of Health.
- In spite of undertaking top academic and administrative posts, it is possible for someone to attain and maintain other skills, for example, in music and writing.

## Introduction

Martin Downer was born on 9 March 1931 in Shrewsbury, Shropshire. His parents were Reginald (Rex) and Eileen (*née* Craig) Downer. His father was a general medical practitioner and an obstetrician at the local Shrewsbury Infirmary.

Martin was educated at Shrewsbury Preparatory School, then, following a common entrance examination, at the main school. The school was founded by King Edward VI in 1552 by Royal Charter. Other alumni include Charles Darwin (naturalist, geologist and evolutionary biologist), Michael Palin (travel writer and TV presenter), Michael Heseltine (politician) and Baron (Martin) Rees (Astronomer Royal and President of the Royal Society).

## And so to dentistry

Martin studied dentistry at Liverpool Dental School and qualified LDSRCS (Licence in Dental Surgery, Royal College of Surgeons of England) in 1958. He initially worked for the Pilkington Glass Company in St Helen's. In 1961, Martin moved to London to work for Haringey Borough Council in Crouch End, North London (part of the Middlesex County school service).

London experienced a massive municipal shake-up when the existing London County Council (LCC) ceased in 1965. Its area was combined with parts of Surrey, Kent and Essex, plus all of Middlesex under a new Greater London Authority. For education purposes, the former LCC area was controlled by a single Inner London Education Authority (ILEA). Education in the new outer London boroughs was run locally. School health, including dentistry and nursing, was part of, and thus financed by, the education departments. The ILEA's Dental Adviser, Ken Webster, had no managerial control over clinics but advised the education authority if it should agree or not to developments and the associated financial expenditure proposed by the boroughs' chief dental officers (CDOs). However, he did manage two mobile surgeries and a dental health education officer, available for use by the boroughs.

Martin became CDO of the Borough of Kensington and Chelsea (K&C) and thus Principal School Dental Officer (PSDO) for the K&C division of the ILEA. Although his contract was with K&C, 90% of his salary and budget was provided by the ILEA. Managing the school dental service, including its clinics, gave Martin a high degree of responsibility, plus managerial and administrative experience. All 13 CDOs/PSDOs of the inner London boroughs and the City of London met weekly to discuss matters of mutual interest, for example, organising dental health education programmes and purchasing mobile clinics for use in schools across all the boroughs. Advice and support were available to Martin from the other CDOs when needed.

By 1968, Martin managed three full-time dental officers and four dental surgery assistants working in four surgeries. His service carried out dental inspections in 41 infants and juniors, three secondary and two nursery schools, covering two-thirds of the primary school children.^1^

Treatment was mostly conservation and some prevention. The Department of Health was then against the latter, suggesting the service did more fillings. CDOs such as Martin had many arguments with the Ministry, continuing with these activities in spite of negative reports to the borough. There were few crowns or inlays. In a time of poor dental health, general anaesthetics were provided in two clinics. An ILEA mobile surgery was occasionally used at schools, with Martin providing the staff. He began to build up an orthodontic service.

Martin was very keen on dental health education, supported by his manager, the Medical Officer of Health, John Weir. Education was both chairside and community, with many dental health education sessions provided for school children. Martin was keen to educate mothers about oral health, especially rampant caries caused by using vitamin syrups in reservoir feeders.

The borough's main health education programme in 1968 was a dental health campaign, with cooperation between the dental and nursing teams. They emphasised four rules: nourishing diet; avoidance of sweet, sticky carbohydrates, especially between meals; regular brushing of teeth and gums; and regular examination by a dentist. Posters were displayed throughout the borough and many leaflets were distributed.

Exhibitions were mounted by health visitors and other nursing staff at two welfare centres. They designed and built the displays and manned the exhibitions, with assistance from Jenny Thomas, the ILEA's dental health education officer. Dental health films were screened at the exhibitions. Talks were given by the Gibbs Oral Hygiene Service's lecturer, Doreen Land, and by Apple Annie, who distributed apples to visiting parties. Pierre the Clown gave talks in schools and taught children to sing ‘we brush our teeth up and down, while getting a visit from Pierre the Clown'. In two weeks, 1,130 children and 400 adults visited the exhibitions. A total of 16 primary schools were visited by Jenny and Doreen. Talks at mothers' clubs were given by Martin and health visitors. There were regular follow-up visits by Jenny throughout the year. Most primary schools received at least one talk. Martin clearly gained a great deal of knowledge and practical skills during his time with K&C.

## Towards dental public health and Manchester

In the 1950s, the Royal College of Surgeons of England considered introducing a diploma in children's dentistry. However, a visit by Professor Alexander MacGregor to Ghana prompted the Faculty of Dental Surgery of the Royal College of Surgeons of England to change its focus to public health dentistry. He explained there was much untreated disease in Ghana. Many dentists came to the United Kingdom (UK) from Africa to study, usually hoping to obtain an LDS. A large proportion of all the candidates failed the examination. In any case, he said the need in Ghana was not for surgeons but for people who could help the many people with routine dental problems needing prevention and simple treatment: a public health approach. As a result, in 1968 the College introduced a Diploma in Dental Public Health (DDPH).

The six London schools cooperated to develop a course of study. The College divided the course requirements into two parts: part one was theory and part two was practical, including epidemiology, methodology, visits to water fluoridation plants and so on. The schools wanted time to develop a good practical programme so said they would initially only take students exempted from part two of the exam, thus attending for 2½ days a week for the theory component. This made it impossible for the very foreign students for whom the exam was originally designed.

A number of UK dentists jumped at the opportunity, including some senior school dental officers (for whom there was then no relevant higher qualification). Martin was one such person. With the agreement of his Health Committee and support of the Medical Officer of Health, he re-jigged his hours to make his attendance possible. The author was a co-student when Martin began his studies in October 1968. He recalls that Martin made a mark on the course, especially with his knowledge of and ability to use statistics, which was of major use to his later work. He often sat with his eyes glued to Neil Chiltern's book on statistics^2^ while everyone else was listening to a lecture. Martin gained the DDPH in July 1969, one of the first to gain the qualification.

In 1970, Martin was appointed by the University of Manchester Dental Health Unit to its second Colgate Research Fellow (after Andrew Rugg-Gunn), working under (later Professor) Phil Holloway, who became a friend as well as a close colleague. Initially, Martin conducted a randomised controlled trial of a supervised school-based fluoride intervention. He then researched the validity of diagnosing caries into dentine, a topic gaining a longstanding interest. His 1974 PhD was titled ‘Aspects of the validity of diagnosis in the epidemiology of dental caries'.

Martin was an excellent administrator and a talented computer programmer. In 1968, when Andrew Rugg-Gunn arrived, Manchester ran an Atlas computer (perhaps then the best in the world). He had to write all the programmes for analysing clinical trials in Atlas Autocode, similar to Algol. Atlas was retired in c1970 and the University bought an IBM, so Andrew had to change all the programmes to Fortran. Martin then joined in. Writing in Fortran 1V, he developed two programmes for the analysis of caries clinical trials and the conduct of treatment needs studies, both used well into the 1980s.

## Reorganisation of the NHS: support for juniors and outreach clinics

Martin was well-qualified for the major 1974 NHS reorganisation. He was appointed Area Dental Officer for the Borough of Salford in Lancashire, one of the newly established posts. The university appointed him as Honorary Senior Lecturer in Community Dentistry and Adviser on Systems Analysis in the Dental Health Unit.

In 1976, Iain Mackie was appointed by Martin as a Community Dental Officer, his first job after graduating from Manchester. Iain had always planned to be a general dental practitioner. However, in his final year, Iain volunteered to join a pilot group of students who left the dental hospital environment to treat children in a school clinic in a deprived area of Manchester. This so affected Iain that he joined the community dental services (CDS). Iain says Martin was a great mentor to his staff and very approachable, encouraging them to undertake further training and qualifications. Thus, Iain gained a DDPH and Master of Science.

Soon after Martin joined Salford, Andy Blinkhorn was appointed as Senior Dental Officer to work in a newly built Churchill Way Clinic in the middle of Salford. Its seven surgeries were specifically designed for outreach teaching of students. Salford became the second area in the country to teach students in an outreach clinic, copying Manchester.^3^

The Dental School's Phil Holloway and Maureen Attrill, Area Dental Officer for Manchester, set up the first pilot clinic in 1974. They needed special dispensation because dental students were then only allowed to treat patients in a hospital environment. Pam Dixon was the supervising teacher.^4^

Martin and Andy were keen that the CDS should be involved in research, so undertook one of the first pragmatic clinical trials to be carried out and published.^5^

He laid the foundations for the rise to fame of Andy Blinkhorn and Iain Mackie. Both became professors and honorary consultants.

## A Scotland interlude

**Fig. 1:**
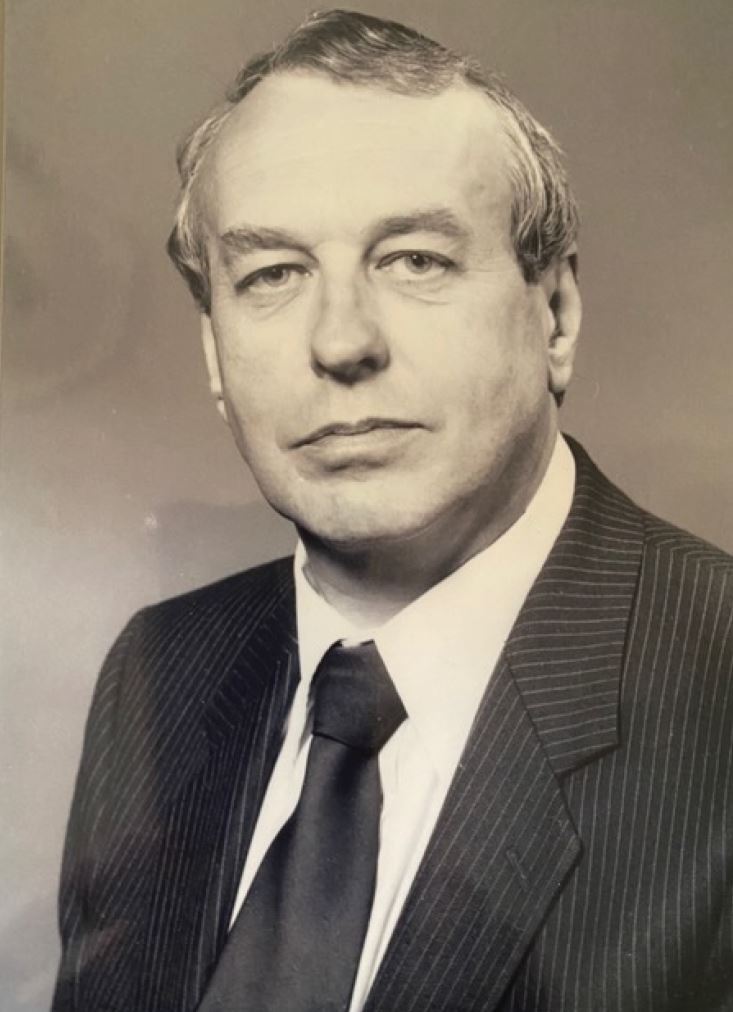
Martin Downer

In 1979, Martin (Fig. 1) was appointed Chief Dental Officer in the Scottish Home and Health Department and became Honorary Senior Lecturer to the Universities of Edinburgh and Dundee. Discovering an existing formal collaborative health agreement with Finland, he organised joint assessments of the oral health of Edinburgh and Helsinki school children. The project went well and was one of the few practical successes of the Finnish-Scottish collaboration. A follow-up study, including the Republic of Ireland, under the direction of Professor Denis O'Mullane, monitored the influence of Ireland's water fluoridation programme. This tri-country study of Scotland, Ireland and Finland was one of the few truly joint European academic successes. Fluoridation remained a major interest while in Scotland.^6^

## Back to England

Martin's talents were increasingly recognised. In 1983, he was appointed CDO to the Department of Health and Social Security in London; later translated to CDO for England in the Department of Health. During that time, the University of Manchester awarded Martin its Doctorate of Dental Surgery for his 1989 thesis ‘Studies in dental epidemiological method and health care evaluation'.

In 1990, he retired from the civil service and became Professor of Oral Health Policy and Head of the Department of Dental Public Health at the Eastman Dental Institute, remaining until 1996. With Ruth Holt, he trained many postgraduates in dental public health, especially those from overseas. This interest in building dental public health capacity internationally was confirmed by his being awarded visiting professorship/lecturer in 11 overseas universities.

Martin was a founder member of the British Association for the Study of Community Dentistry (President 1998-1999), European Association of Dental Public Health, and International Academy of Oral Oncology (2005). Some other commitments are shown in Box 1.

Box 1 Some additional roles undertaken by Martin Downer

- Member of General Dental Council (1979-90; Chair of Dental Health Education Sub-committee 1979-82)
- Member of Scottish Health Service Planning Council (1979-83)
- Member of Dental Strategy Review Group (1980-81)
- Member of Joint Consultants Committee (1983-90)
- Member of WHO Expert Advisory Panel on Oral Health (1984-99)
- Chair of Adult Dental Health Survey Steering Group (1987-88)
- Chair of DH Committee on Continuing Education and Training (1988-90)
- Chair of UK Working Group on Screening for Oral Cancer (1991-93)
- Member of Higher Education Funding Council for England Research Assessment Exercise, Clinical Dentistry Panel (1995)
- Member of NHS Primary Dental Care R&D Programme, Peer Review Panel (1997)
- Member of Advisory Scottish Committee on Dental Establishments, SHHD
- Chair of DHSS Central Manpower Committee's Dental Sub-committee

## Research activities

Martin carried out a wide range of research. With colleagues, he carried out several reviews to aid the development of UK and foreign dental policy. In 2005, they examined evidence of good public health practice to define policies to improve oral health in the European Union and associated countries.^7^ They also examined available European data on oral health, possible determinants and their dental services. Finally, they suggested strategies for improving oral health service policy in Europe.

Another review considered what research was needed related to water fluoridation.^8^ After examining information from a conference on ‘Researching water fluoridation: evaluation and surveillance' at Manchester University, they synthesised ideas for future research and public health surveillance. Together with information from other sources, they constructed a framework for developing fluoridation research/surveillance.

They concluded that covering a full range of criteria for comprehensive evaluation and surveillance would be too complex and costly. Instead, they suggested a piecemeal strategy: pilot studies and a series of separate but linked projects.

Martin was very interested in oral cancer screening. He collaborated with Paul Speight, Professor in Oral and Maxillofacial Pathology in Sheffield, to improve the public health aspects of oral cancer screening. Speight said^9^Martin made major and seminal contributions to research into oral cancer screening, which ultimately informed national and international policy. In 1993, they set up a UK working group for screening of oral cancer and pre-cancer and published a special edition of *Community Dental Health*.^10^ From this, a UK and then an international movement started to investigate the pros and cons of oral cancer screening. Their group published more articles than any others worldwide: over 20 peer-reviewed papers, mostly primary research or systematic reviews.

Downer and Speight's work had an international reach, with many conferences worldwide. In December 2000, they were invited to a specialist workshop on screening for oral cancer at National Institute of Dental and Craniofacial Research (NIDCR), USA. The evidence from their work and others (mainly an Indian group) informed the Unites States and UK Governments. The UK National Screening Committee and the USA's National Cancer Institute, NIDCR and the Preventive Services Task Force have not recommended oral cancer screening, not because of cost, but to the natural history of the disease and inadequacy of available screening tests, with very high false positives.

Paul Speight emphasised that Martin's leadership and contribution to the group was essential: Paul provided the pathology and clinical input, Martin the broader perspective - especially statistical expertise and qualitative research methods.

Martin led on a seminal series of papers that explored the pros and cons of screening.^11^^,^^12^From them emerged a large project to undertake a detailed analysis of the benefits and cost-effectiveness of oral cancer screening. Funded by the UK Health Technology Assessment Programme, they produced a monograph that is highly cited and used worldwide to inform funding decisions and project design.^13^ That was probably Martin's final dental publication.

Martin's other publications are too numerous to list here. However, a few illustrate the breadth of his work. His research and writing abilities showed early, with papers on caries and periodontal disease in girls of several ethnic groups^14^ and an evaluation of oral hygiene practices.^15^Interestingly, one 2011 paper was written in German.^16^

Together with some Manchester colleagues, he took an interest in examining historical evidence of early oral disease.^17^

Martin often analysed methods of data recording, for example, of caries. He often worked with researchers in other schools, both abroad as well as in the UK.^18^ Based in Salford, Martin was able to compare health and needs of local children with those in fluoridated Birmingham.^19^

Unsurprisingly, in 2010, Martin was awarded the International Association for Dental Research's Distinguished Scientist Award in Behavioural, Epidemiologic and Health Services Research.

## Dental publications

Martin enjoyed writing. He had over 100 publications in peer-reviewed journals, including the *British Dental Journal*, *The Lancet, Community Dental Health* and *Oral Oncology*.

He authored or contributed chapters to some 20 text or reference books, including:

- M. C. Downer, S. Gelbier, D. E. Gibbons, J. E. Gallagher. *Introduction to dental public health.* Switzerland*:* FDI World Dental Press, 1994 (also translated into Japanese)
- ‘Principles of screening - oral cancer'. *In* P. S. Rothwell (ed) *Pathways in practice, vol II: primary dental care*. London: Faculty of GDPs (UK), 1997
- ‘Oral cancer'. *In* C. M. Pine, R. Harris (eds) *Community Oral Health.* 2nd ed. Copenhagen: Quintessence, 2005.

Martin was editor of *Community Dental Health*, the journal of the British Association for the Study of Community Dentistry, from 1993-2002, overseeing its enlargement to A4 size.

He was also a contributor to the Oxford Dictionary of National Biography.^20^

## The family and music

Martin married Anne Evans, an educational psychologist, in London in October 1961. They remained married for 56 years, with four daughters (Stephanie, Caroline, Diana and Gabrielle) and 13 grandchildren. Caroline (Drugan: BDS 1987; PhD 1997; FDS 1992; MSc 1992; MPH 2003) studied dentistry in Bristol and then followed Martin's footsteps into dental public health. They co-authored a number of papers.

At first meeting, Martin sometimes seemed aloof, but in fact, he was known for his conviviality, kindness and zest for life, including a joy for food. In his youth, Martin spent much time in jazz clubs. With friends, he showed a great sense of humour and was great fun on a night out. Colleagues attest to many laughs in bars and restaurants on their travels.

Among his earliest hobbies was playing the clarinet, learned at school, and later the saxophone. He played in a semi-pro jazz band throughout his student days at local venues, most frequently Liverpool's Cavern Club. In fact, Martin was in a band which played at the opening night of the Cavern Club in 1957.^21^ One claim to fame was that The Beatles once played as their interval band. The club became a centre of jazz and rock and roll, closely associated with Merseybeat and regularly played host to The Beatles in their early years.

**Fig. 2:**
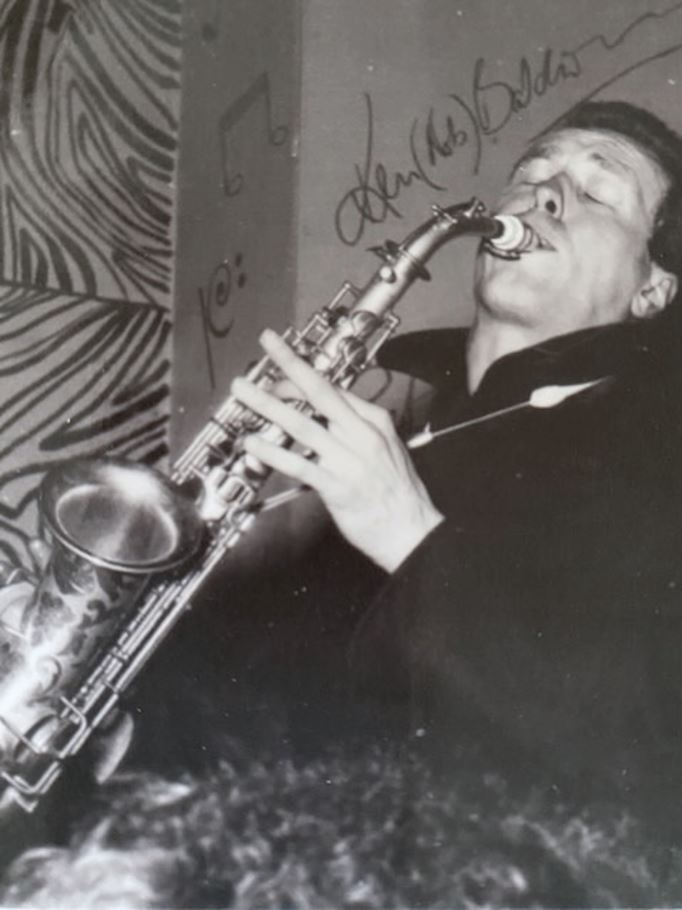
Martin playing on his saxophone

After graduation, Martin played clarinet and saxophone (Fig. 2) professionally for a few years, mostly in London clubs with Charlie Galbraith's All Star Jazz Band and the London City Stompers. He broadcast and gigged with Mick Mulligan's Magnolia Jazz Band and its featured vocalist, George Melly. Martin made several records and managed a top 20 hit called ‘Too many beautiful girls' and even appeared on TV's *Top of the Pops*. The All Star Jazz Band is available on Spotify for those interested to hear Martin playing his saxophone.

## Literary output

In retirement, Martin studied creative writing at Bath Spa University (MA, 2010). As Martin Craig-Downer, he wrote several novels and a selection of short stories published by Mardi Books: *The tank room* (2011), *Over there* (2012) and *Scenes behind the power* (2014). He also assisted in editing and contributed three very diverse short stories to an anthology: *Unexpected tales from the ends of the earth* (2012).

*The tank room,* set in 1950s Liverpool, features his love of jazz, with musicians rubbing shoulders with up-and-coming artists, writers and poets. Also a man's love for two women from very different backgrounds: a promiscuous and precocious teenager and a wayward daughter of landed gentry.^22^

*Over there* follows three groups of individuals from World War II into the twenty-first century: a provincial English family, striving Italian-American immigrants and a mafiosa family, with unexplained deaths.

## Non-recognition

It is notable that Martin was never given a national award. Apart from anything else, he was probably the UK's longest serving CDO (Scotland and England). Although a travesty, in a way, perhaps it was a tribute to Martin - he constantly challenged the government on policy rather than just carrying out their wishes. There was much conflict between the government and profession in the late 1980s over funding and the contract for general dental services. A new contract was introduced in 1990 which the profession didn't like. Although its CDO, Martin did not champion the government's stance. Add that to his enormous research contribution, it clearly points to a major national omission.

Martin Downer died on 28 April 2017.
